# Disequilibrium evolution of the Fructose-1,6-bisphosphatase gene family leads to their functional biodiversity in *Gossypium* species

**DOI:** 10.1186/s12864-020-6773-z

**Published:** 2020-06-01

**Authors:** Qún Gě, Yànli Cūi, Jùnwén Lǐ, Jǔwǔ Gōng, Quánwěi Lú, Péngtāo Lǐ, Yùzhēn Shí, Hǎihóng Shāng, Àiyīng Liú, Xiǎoyīng Dèng, Jìngtāo Pān, Qúanjiā Chén, Yǒulù Yuán, Wànkuí Gǒng

**Affiliations:** 1 College of Agriculture, Engineering Research Centre of Cotton of Ministry of Education, Xinjiang Agricultural University, Urumqi, China, 311 Nongda East Road, Urumqi, 830052 China; 2 State Key Laboratory of Cotton Biology, Institute of Cotton Research, Chinese Academy of Agricultural Sciences, Anyang, China; 3 Research Base, State Key Laboratory of Cotton Biology, Anyang Institute of Technology, Anyang, China; 4 Zhengzhou Research Base, State Key Laboratory of Cotton Biology, Zhengzhou University, Zhengzhou, China

**Keywords:** Cotton, Fructose-1, 6-bisphosphatase, Evolution, Translocation, Expression patterns

## Abstract

**Background:**

Fructose-1,6-bisphosphatase (FBP) is a key enzyme in the plant sucrose synthesis pathway, in the Calvin cycle, and plays an important role in photosynthesis regulation in green plants. However, no systemic analysis of FBPs has been reported in *Gossypium* species.

**Results:**

A total of 41 *FBP* genes from four *Gossypium* species were identified and analyzed. These *FBP* genes were sorted into two groups and seven subgroups. Results revealed that *FBP* family genes were under purifying selection pressure that rendered FBP family members as being conserved evolutionarily, and there was no tandem or fragmental DNA duplication in *FBP* family genes. Collinearity analysis revealed that a *FBP* gene was located in a translocated DNA fragment and the whole *FBP* gene family was under disequilibrium evolution that led to a faster evolutionary progress of the members in *G. barbadense* and in A_t_ subgenome than those in other *Gossypium* species and in the D_t_ subgenome, respectively, in this study. Through RNA-seq analyses and qRT-PCR verification, different *FBP* genes had diversified biological functions in cotton fiber development (two genes in 0 DPA and 1DPA ovules and four genes in 20–25 DPA fibers), in plant responses to *Verticillium* wilt onset (two genes) and to salt stress (eight genes).

**Conclusion:**

The FBP gene family displayed a disequilibrium evolution pattern in *Gossypium* species, which led to diversified functions affecting not only fiber development, but also responses to *Verticillium* wilt and salt stress. All of these findings provide the foundation for further study of the function of *FBP* genes in cotton fiber development and in environmental adaptability.

## Background

Fructose-1,6-bisphosphatase (FBP, EC 3.1.3.11) catalyzes the decomposition of fructose 1,6-diphosphate (F-1,6-P_2_) into 6-phosphate fructose (F-6-P) and inorganic phosphorus (Pi) [1, 2]. It is ubiquitous across organisms and is a key enzyme in the Calvin cycle and the gluconeogenesis pathway [3, 4]. These reactions are involved in carbon fixation and sucrose metabolism and are present in the chloroplast stroma and the cytosol of green plants [5]. In most higher plants, FBP exists in three possible forms including a monomer, dimer, and tetramer, among which only the tetramer has catalytic activity [6].

In higher plants, based on their different catalytic mechanisms and independent evolutionary phylogensis, FBPs can be classified into two groups, cytosolic FBPs (cyFBPs) and chloroplast FBPs (cpFBPs). cyFBP plays an important regulatory role in the gluconeogenesis pathway and the synthesis of sucrose, while cpFBP is involved in the reduction of the pentose phosphate pathway [3, 4, 7]. cyFBP and sucrose phosphate synthase (SPS) are the main rate-limiting enzymes in the sucrose synthesis pathway [8]. 6-phosphate fructose is an essential monosaccharide for sucrose synthesis, and cyFBP and 6-phosphate fructokinase (PFK), pyrophosphate, and 1,6-diphosphate fructose transferase (PFP) jointly regulate the formation of fructose-6-phosphate. cyFBP can be inhibited by the metabolic product AMP, 2,6-diphosphoric fructose (F-2,6-P_2_), and also by Mg^2+^ and Ca^2+^, while cpFBP is not sensitive to either AMP or fructose-1,6-diphosphate [2]. Studies of FBP over expressions show that it can increase photosynthetic capacity, sucrose synthesis, and promote sugar accumulation, thereby accelerating plant growth.

Recently, some *FBP* genes have been cloned in several species such as *Beta vulgaris*, *Spinaciaoleracea*, *Glycine max*, *Arabidopsis thaliana*, *Pisumsativum*, *G. hirsutum* and *Pyropia haitanensis*, and other plants [5, 9–14]. The main research activities on these *FBP* genes have included identifying their functions in plant photosynthesis and glucose metabolism through molecular bioinformatic analysis and over-expression [11, 13–18]. In a transgenic study in *A. thaliana*, antisense transcripts were applied to inhibit the expression of a *cyFBP* gene. The decreased expression of the *FBP* gene resulted in decreased sucrose synthesis, accumulated intermediate metabolites, and eventually blocked photosynthesis [11]. In another study in *A. thaliana*, over-expression of a *cyFBP* gene caused an increase in sucrose synthesis and promoted plant growth in transgenic plants [15]. Inhibiting the expression of this gene in *Solanum tuberosum* could also reduce sucrose synthesis during the photosynthetic process [16]. In rice, loss of *cyFBP* reduced photosynthetic sucrose synthesis and delayed plant growth [17]. When *cpFBP* was inhibited in tomato, only small changes in carbohydrate metabolism were observed, but this inhibition caused a significant decrease in fruit size [18]. The different response modes of *PhcpFBP* mRNA levels in *Pyropia haitanensis* indicated that *cpFBP* also plays an important role in response to abiotic stresses such as high temperature and drought [14]. The different expression level of *GhFBP* at different times during cotton fiber development indicated that it plays a key role in the early stage of fiber secondary cell wall development [13].

Cotton is an important economic crop in the world, and cotton fiber is an important raw natural material for the textile industry. Cotton fiber is developed from the differentiation of a single ectodermic epidermal cell, and the fiber formation process can be divided into four distinct but partially overlapping periods: initiation, elongation (primary wall formation), secondary wall thickening, and dehydration maturity [19]. Many methods, including QTL identification [20–22], GWAS analysis [23–26], and functional gene identification [27–29], have been used to tackle the problems of fiber development and fiber quality formation. Studies have revealed that fiber development is a very complex process, with a large number of metabolic pathways providing material support, and thousands of specific genes being involved in expression regulation. At the same time, *Verticillium* wilt, which has the nickname “cotton cancer,” is currently one of the most serious diseases that restricts cotton production and affects fiber quality [30]. A high concentration of saline stress also negatively [31] affects the growth, development, and fiber quality of cotton [32, 33].

Although a few functional studies of *FBP* genes in some plant species have revealed that *FBP* genes could have certain impacts on various biological activities, *FBP* behavior is still poorly understood. Specifically, how *FBP* genes function at the whole genome level, especially in *Gossypium* species, remains unclear. The completion of whole genome sequencing databases for two important diploid cotton species *G. raimondii* [34, 35] and *G. arboreum* [36], and two domesticated tetraploid species *G. hirsutum* [37–40] and *G. barbadense* [39–41], provides brand-new platforms for functional genomic studies. In this study, we identified 41 *FBP* family members in the genomes of these four cotton species and 73 *FBP* members in nine other species. Intensive bioinformatic analyses, including physicochemical properties, chromosomal localization, evolutionary relationships and gene structure, conserved motifs and *FBP* domain features, and functional expression analyses including transcriptomic and quantitative RT-PCR (qRT-PCR) were performed. The results indicated that *FBP* genes were involved in plant responses to biotic and abiotic stresses, as well as cotton fiber formation. This study provides a foundation for functional verification of the *FBP* genes of cotton in the future and useful information for the improvement of cultivars with excellent fiber quality and broad environmental adaptability.

## Results

### Identification of FBP family members

A total of 41 *FBP* genes from four *Gossypium* species, including 14 in *G. hirsutum* (*GhFBP*), 15 in *G. barbadense* (*GbFBP*), 6 in *G. arboreum* (*GaFBP*), and 7 in *G. raimondii* (*GrFBP*), were identified in this report (Supplementary file 1). The number of *FBP* genes in the tetraploid genomes of *G. hirsutum* and *G. barbadense* (AD genome) was almost double those in the diploid genomes *of G. raimondii* (D genome) and *G. arboreum* (A genome). These two tetraploid *Gossypium* genomes arose from a natural hybridization between two ancestors of diploid *G. raimondii* and *G. arboreum* [38, 40, 42].

In addition, in order to elucidate the evolutionary and phylogenetic relationship of these *FBP* genes, we identified 73 *FBP* family genes in nine other species, including 4 in *Arabidopsis thaliana*, 5 in *Theobroma cacao*, 12 in *populus trichocarpa*, 12 in *Glycine max*, 11 in *Zea mays,* 5 in *Vitis vinifera*, 6 in *Selaginella moellendorffii,* 11 in *Physcomitrella patens,* and 7 in *Oryza sativa* (Supplementary file 1).

### Phylogenetic analysis of the FBP gene family

**Fig. 1:**
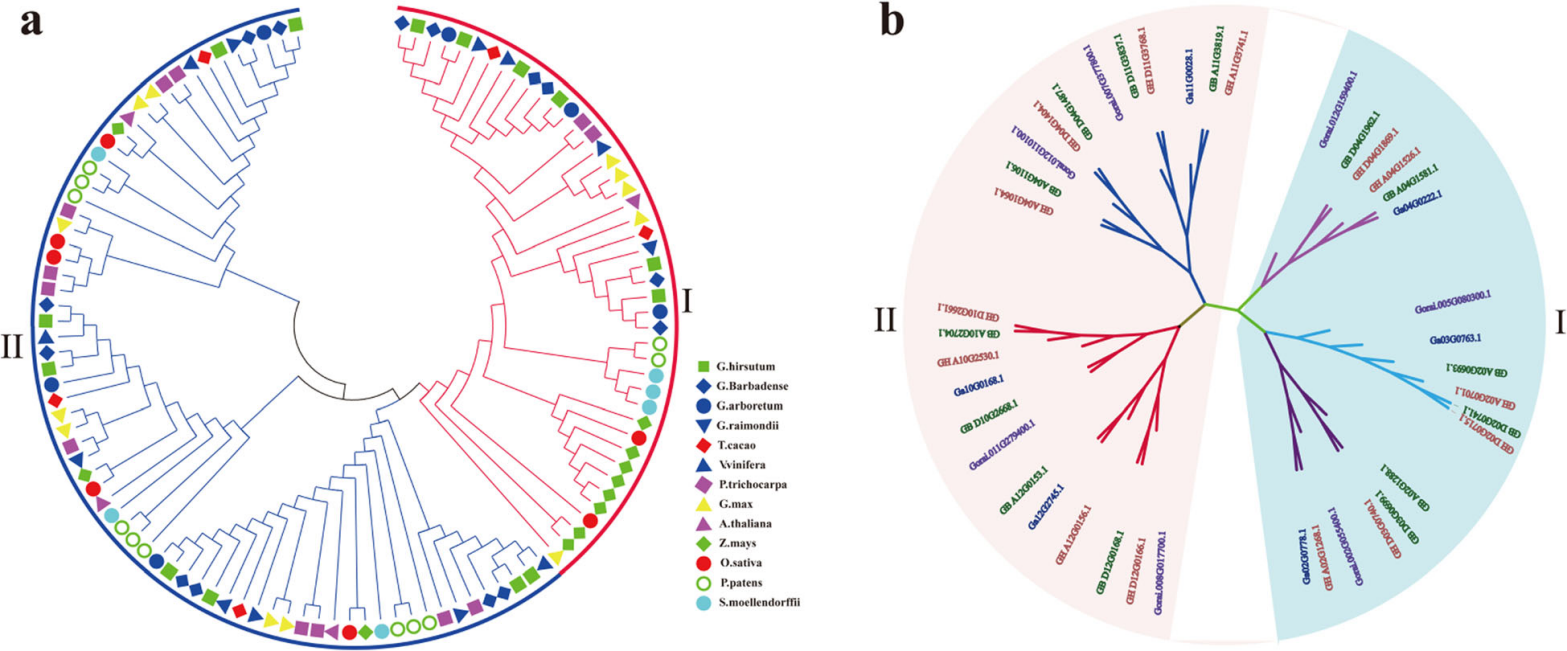
Phylogenetic trees of *FBPs*. **a** Phylogenetic tree of 114 *FBPs* from 13 species, including *G. hirsutum*, *G. barbadense*, *G. arboreum*, *G. raimondii*, *A. thaliana*, *T. cacao*, *P. trichocarpa*, *G. max*, *Z. mays, V. vinifera*, *S. moellendorffii, P. patens* and *O. sativa*; **b** Phylogenetic tree of 41 *FBPs* from four *Gossypium* species. I represent *cyFBPs* and II represent *cpFBPs*

To elucidate the evolutionary relationship of the identified FBP proteins between *Gossypium* and other species, the amino acid sequences of all the FBP proteins were aligned to identify their phylogenetic similarities with orthologs using the neighbor-joining model from MEGA 7, and a phylogenetic tree was thus constructed as shown in Fig. 1a. According to their evolutionary relationships, 114 FBP proteins were divided into 2 groups: cytosolic FBPs, (*cyFBPs*) which included 40 members; and chloroplast FBPs, (*cpFBPs*) which included 74 members [7, 14]. The result of phylogenetic analysis indicated that FBPs had a closer evolutionary relationship between the four *Gossypium* species as compared with other species. The phylogenetic results also indicated that between all the other species, cocoa had the closest evolutionary relationship to the examined cotton species [38, 40]. Further phylogenetic analysis of *FBPs* from the four cotton species indicated that the *cyFBPs* were assorted into three subgroups, while the sorted into *cpFBPs* four subgroups (Fig. 1b). Each subgroup of *Gossypium FBPs* consisted of six members, including one from the A genome (*G. arboreum*) and one from the D genome (*G. raimondii*), two from *G. hirsutum*, and two from *G. barbadense*. As both of *G. hirsutum* and *G. barbadense* are comprised of A_t_ and D_t_ subgenomes, each subgenome provided one member in each sub-group of the *FBP* family. There is only one subgroup in cpFBPs that had 5 *FBPs*, but there was no *FBP* from *G. arboreum* identified in these analyses (Fig. 1b).

### Gene structure and protein domain of FBP family members

The length of amino acid (aa) sequences of FBP proteins ranged from 341 to 608, 341 to 412, 341 to 428, and 341 to 606 in *G. arboreum*, *G. raimondii*, *G. hirsutum*, and *G. barbadense*, respectively. The *cyFBP* group had 18 members (42.87%), which had a uniform length of 341 aa with only two exceptions, namely Gorai.005G080300.1 and GB_A02G1288.1. The *cpFBP* group had 23 members (57.13%), which had a varied length of aa sequences (Fig. 1, Supplementary file 2). The PI values of the four cotton FBPs ranged from 5.00 to 7.68.

**Fig. 2:**
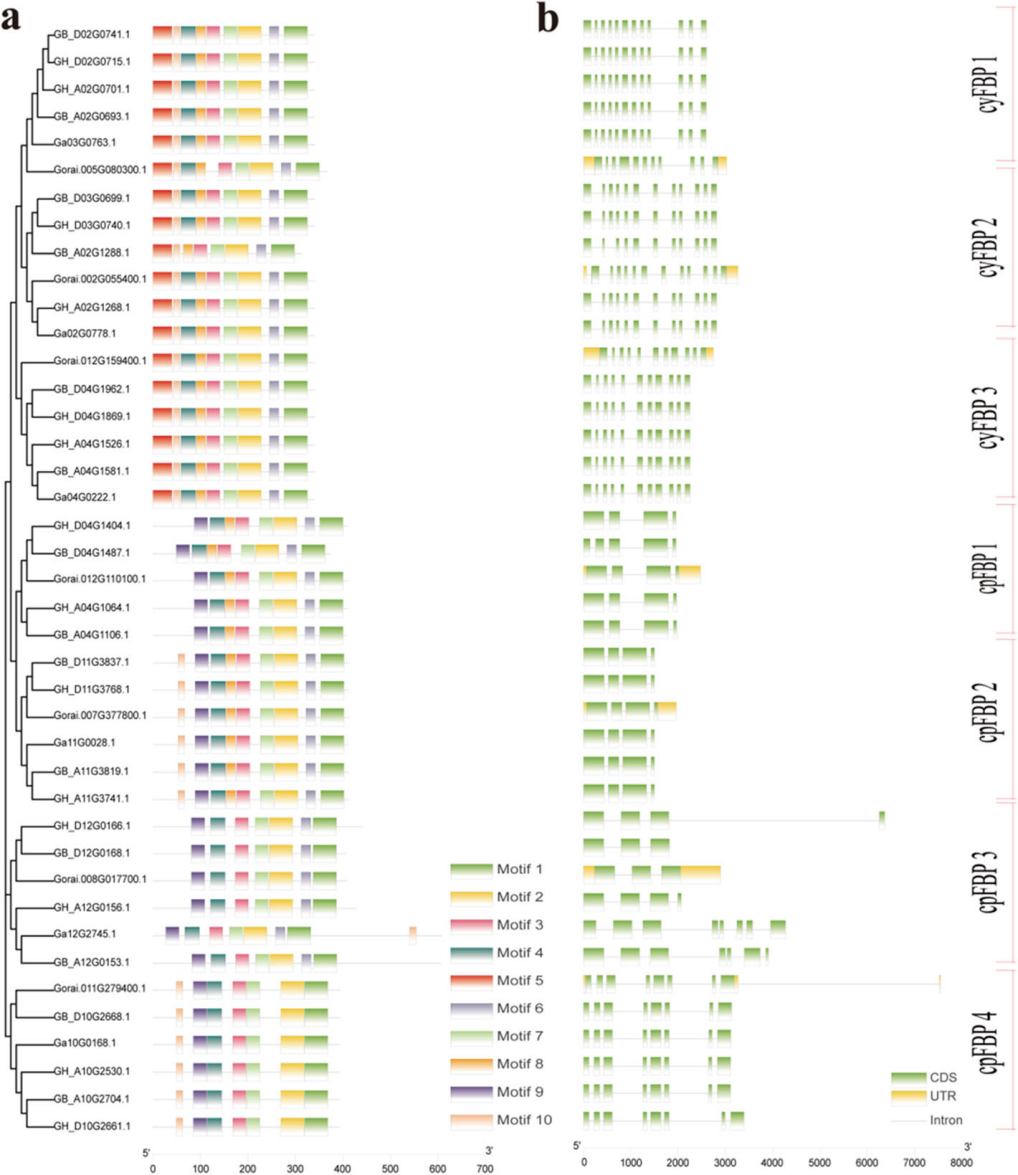
Phylogenetic relationship, motif and gene structures of *FBP* members in four cotton species

In total, 10 motifs were identified in the FBP family in the four *Gossypium* species, with each FBP containing 7 to 9 motifs in general (Fig. 2a, Figure S1). The significant difference between *cyFBPs* and *cpFBPs* was that motif 5 was identified exclusively in *cyFBPs,* while motif 9 was exclusively present in *cpFBPs*. Each phylogenetic subgroup had a similar composition and arrangement of motifs, which was highly consistent with the results of phylogenetic analysis. The results also showed some minor variance in motif composition and arrangement between the subgroups (Fig. 2a).

Gene structure analysis also showed consistent results to our phylogenetic and protein motif analyses (Fig. 2b). The exon number of FBP genes ranged from 3 to 12. *cyFBPs* had 11 to 12 exons, while *cpFBPs* only had 3–5 exons. The gene structure of each subgroup was almost the same, which indicated conserved evolution patterns for *FBP* family members. The *cyFBP* gene structures could be further divided into three types (Fig. 2). Both subgroups *cyFBP* 1 and *cyFBP* 2 had 12 exons and 11 introns, with a varied distribution between them. Subgroup *cyFBP* 3 had 11 exons and 10 introns. In contrast to *cyFBPs*, *cpFBPs* had much fewer exons. The *cpFBP* genes could be sorted into four subgroups. Subgroups *cpFBP* 1 and *cpFBP* 2 had 4 exons and 3 introns, with different distributions between them. Subgroup *cpFBP* 4 had 8 exons and 7 introns, while subgroup *cpFBP* 3 had a varied number of exons and introns, and the exon number of this subgroup ranged from 3 to 8. The results also indicated that only FBP genes from *G. raimondii* had UTR structures. This indicated that *cyFBPs* had more complicated gene structures than *cpFBPs* had.

### Analysis of *cis*-acting elements in the promoter regions of homologous FBP genes

**Fig. 3:**
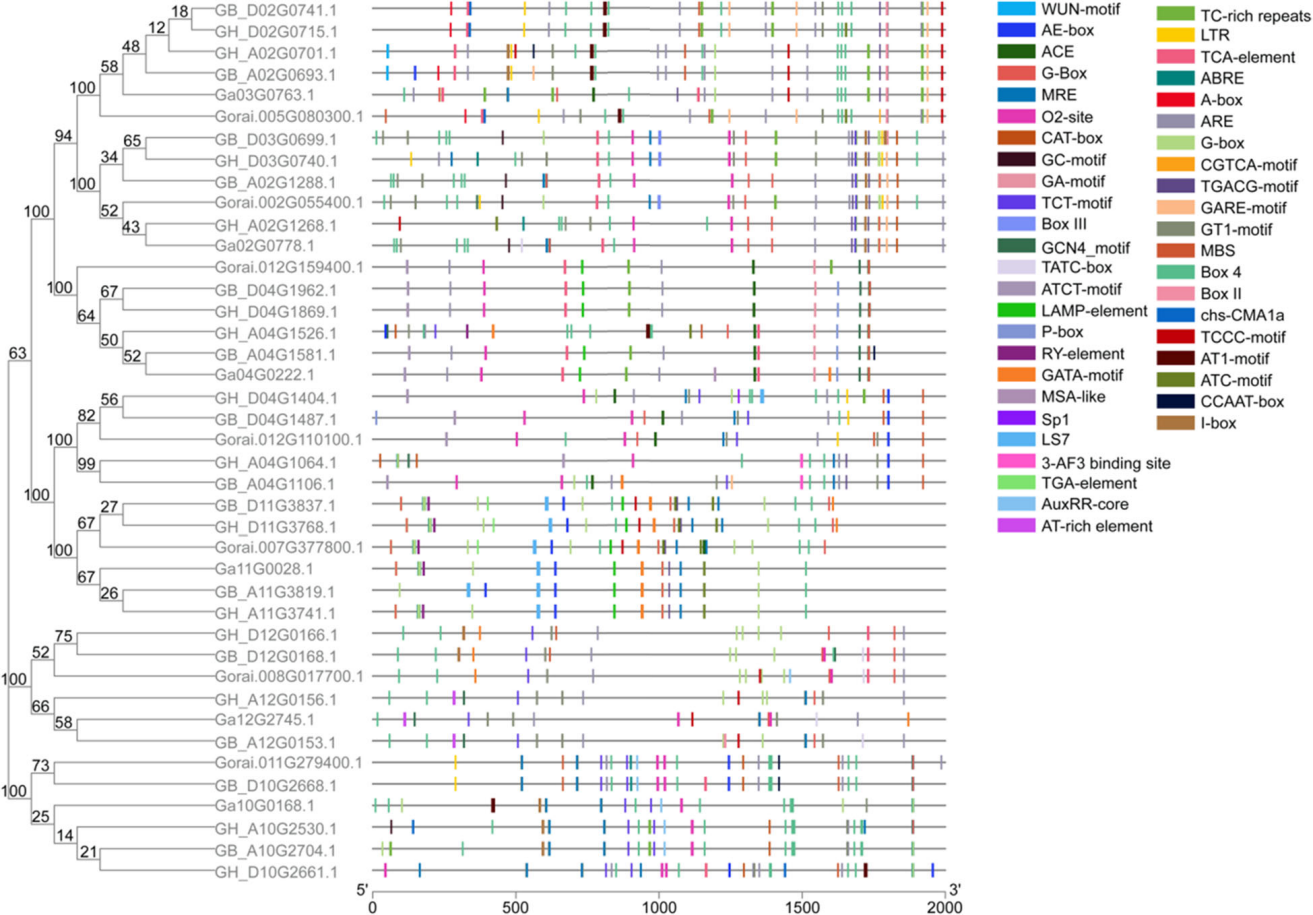
*cis*-acting element analysis of cotton *FBP* genes.

To further understand how *FBP* genes function, the composition and distribution of *cis*-regulatory elements (CRE) were identified in the 5′ untranslated regions 2000 bp upstream of each gene from the PlantCare website (Fig. 3). The results indicated that the composition and distribution of CREs varied significantly across the whole *FBP* gene family. It also could be seen that the CREs had a high congruency with the results of gene structure, protein domain, and phylogenetic analyses. Each subcategory of *FBP* genes had identical or similar compositions and distributions of CREs in their 5′ upstream regions (Fig. 3).

Further analysis indicated that the 5′ up-stream regions of FBP genes contained almost all of the following categories of CREs: constitutive, inducible and tissue-specific. The constitutive CREs include typical basic components such as TATA-Boxes and CAAT-Boxes. Inducible CREs included photo-responsive elements, ATCC-motifs, Box 4, I-Boxes, Sp1, TCCC-motifs, GAG-motifs, gibberellin response elements (GARE-motifs), P-Boxes, abscisic acid responsive elements (ABREs), salicylic acid reaction elements, TCA-elements, anaerobic induction elements (AREs), stress-responsive elements, TC-rich repeats, and MYB binding site (MBS). In addition, the GARE-motif was exclusively identified in the promoter region of one subcategory of genes including *GH_A02G0701.1*, *GH_D02G0715.1*, *GB_A02G0693.1*, *GB_D02G0741.1*, *GH_A02G1268.1*, and *GB_A02G1288.1*.

### Distribution and collinearity analysis of the *FBP* gene family *Gossypium* species

In the genome of *G. arboreum*, *FBP* genes were identified on chromosomes A02, A03, A04, A10, A11, and A12, while in the genome of *G. raimondii*, *FBP* genes were identified on chromosomes D02, D05, D07, D08, D11, and D12. In the tetraploid genomes of *G. hirsutum* and *G. barbadense*, *FBP* genes had similar distribution on chromosomes A_t_02, A_t_04, A_t_10, A_t_11, A_t_12, D_t_02, D_t_03, D_t_04, D_t_10, D_t_11, and D_t_12. Homologous analysis indicated that a homologous gene identified on A03 of *G. arboreum* was identified on chromosome A_t_02 in *G. hirsutum* and *G. barbadense*.

**Fig. 4:**
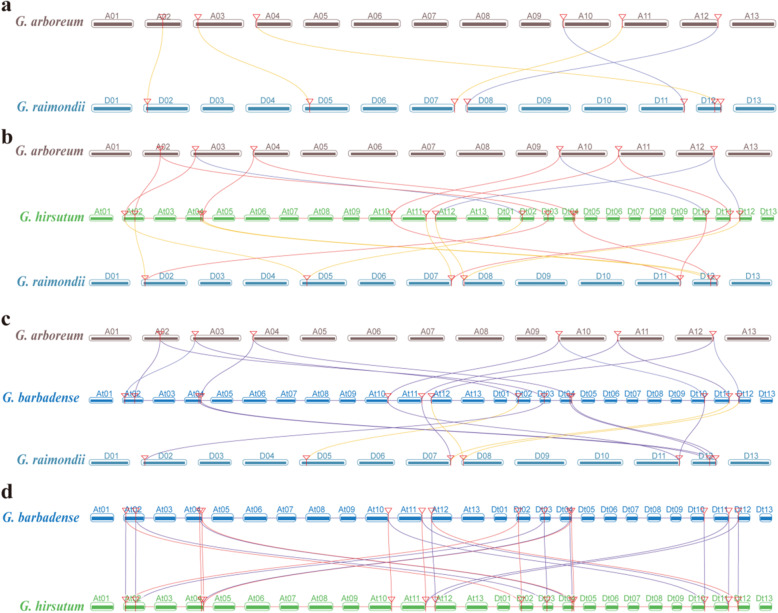
Collinearity of *FBP* genes between different cotton species. **a** collinearity between *G. raimondii* and *G. arboreum*; **b** collinearity between *G. raimondii*, *G. arboreum* and *G. hirsutum*; **c** collinearity between *G. raimondii*, *G. arboreum* and *G. barbadense*. **d** collinearity between *G. hirsutum* and *G. barbadense*

Tandem and fragmental DNA duplication provides major forces that drive the formation of gene families [43, 44] as well as whole genome evolution. In the current study, the duplication events of cotton *FBP* genes were analyzed. Although the results did not support any tandem repeat events occurring during the evolution of the cotton *FBP* gene family, collinearity analysis showed that in these two diploid species the *FBP* genes were perfectly chromosome-pair-wise homologous (Fig. 4a). Meanwhile, in the two tetraploid species, each *FBP* gene from one species (*hirsutum* or *barbadense*) had two homologous genes in both the A_t_ and D_t_ subgenomes in its counterpart species (*barbadense* or *hirsutum*) (Fig. 4d). Collinearity analysis between diploid and tetraploid species indicated that in *G. hirsutum* each gene had two homologous genes in the two diploid species (Fig. 4b), while in *G. barbadense*, two *FBP* genes on GbA_t_02 did not have homologous genes in *raimondii* and one *FBP* gene at GbD_t_12 did not have a homologous gene in *arboreum* (Fig. 4c).

### Analysis of selection pressure of *FBP* genes in four cotton species

**Fig. 5:**
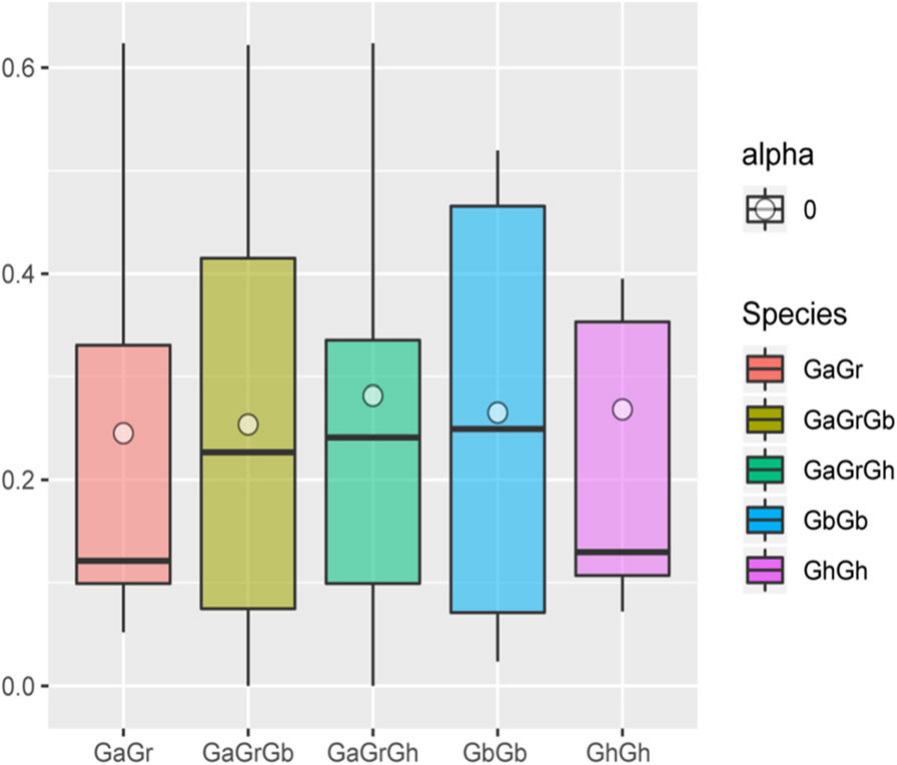
Multiple comparison of Ka/Ks ratios of genes pairs in four *Gossypium* species

Calculating non-synonymous (Ka) and synonymous (Ks) substitution rates is a useful method for assessing sequence variation of protein orthologous in different species or taxa with unknown evolutionary states [45]. The value of Ka/Ks represents the ratio between Ka and Ks of two homologous protein-coding genes. Ka/Ks > 1 indicates that a gene has been positively selected, while a Ka/Ks = 1 indicates that a gene has been neutrally selected, and a Ka/Ks < 1 indicates that a gene has been selectively purified [45]. The Ka/Ks values of homologous *FBP* genes between *G. arboreum* and *G. raimondii* ranged from 0.05 to 0.62, while those between *G. hirsutum* and *G. arboretum* or *G. raimondii* ranged from 0 to 0.8. Those between *G. barbadense* and *G. arboreum* or *G. raimondii* ranged from 0 to 0.6*,* and the values between A_t_ and D_t_ paralogous genes in *G. hirsutum* and *G. barbadense* ranged 0.07 to 0.76 and 0.02 to 0.52, respectively (Fig. 5, supplementary file 3). These results indicated that the *FBP* genes in these four *Gossypium* species were under purifying selection.

### FBP gene expression in fiber development and in response to biotic and abiotic stresses

**Fig. 6:**
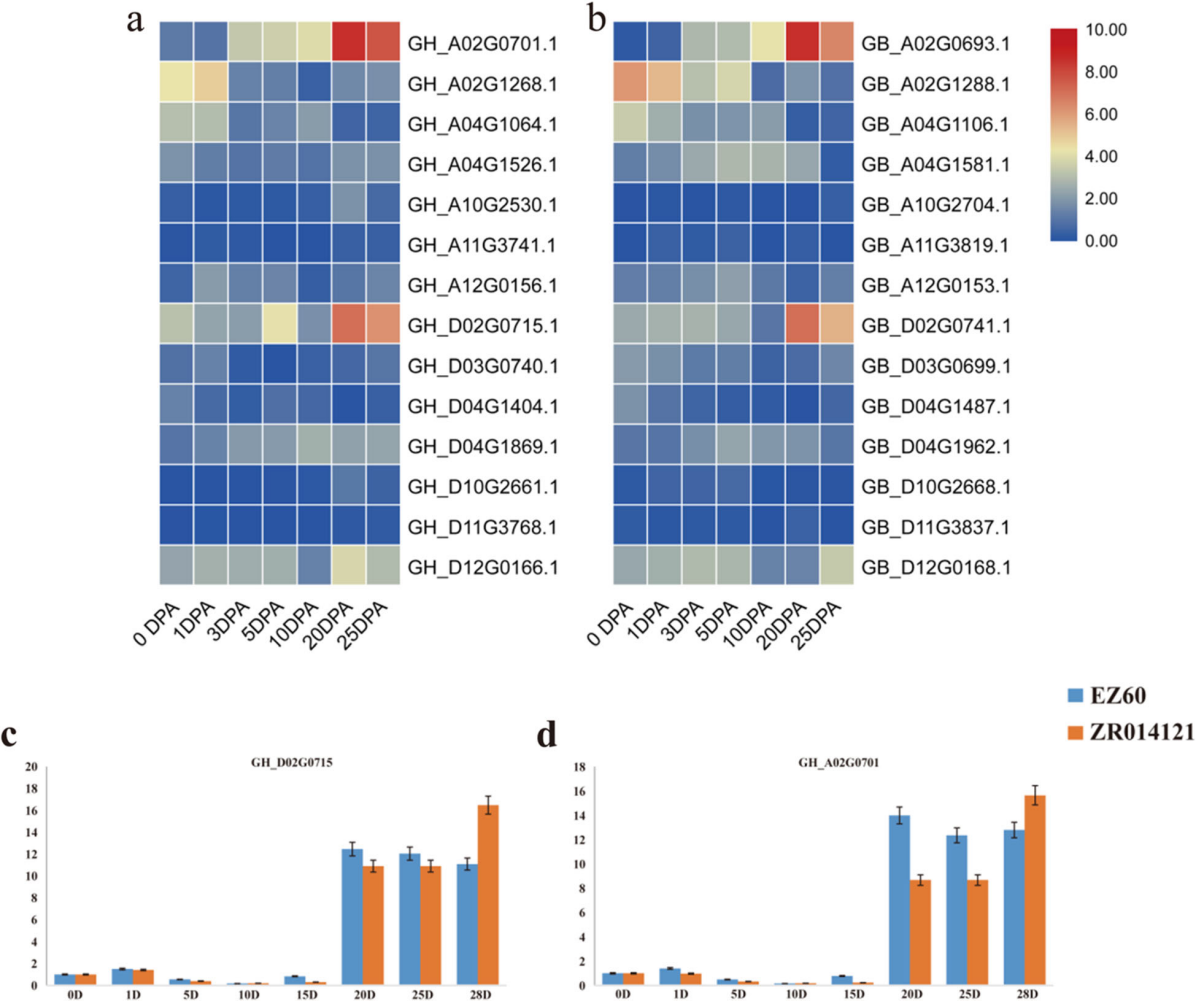
Expression levels of *FBP* genes in different stages of cotton fiber development. **a** Transcriptome analysis of *FBP* genes expression levels during fiber development in *G. hirsutum*; **b** Transcriptome analysis of *FBP* genes expression levels during fiber development in *G. barbadense*; **c** qRT-PCR validation of *GH_D02G0715.1*; **d** qRT-PCR validation of *GH_A02G0701.1*

To explore the potential function of *FBP* genes in the growth and development of cotton fibers, we downloaded cotton fiber transcriptome data from the NCBI SRA database and reanalyzed the expression profiling of *FBP* genes. The results of *FBP* gene expression analysis showed that the homologous genes *GH_A02G0701.1* and *GH_D02G0715.1* from *G. hirsutum*, and *GB_A02G0693.1* and *GB_D02G0741.1* from *G. barbadense* had higher FPKM values in developing fibers at 20 days post-anthesis (DPA) and 25 DPA (supplementary file 4). The homologous genes *GH_A02G1268.1* and *GB_A02G1288.1* had high expression FPKM values in the early stage of the fiber development (0 DPA and 1 DPA ovule) (Fig. 6a, b). The expression of *GH_D02G0715.1* and *GH_A02G0701.1* in the secondary cell wall synthesis stage of fiber development through qRT-PCR validation assays were consistent with in silico transcriptome analysis (Fig. 6c, d).

**Fig. 7:**
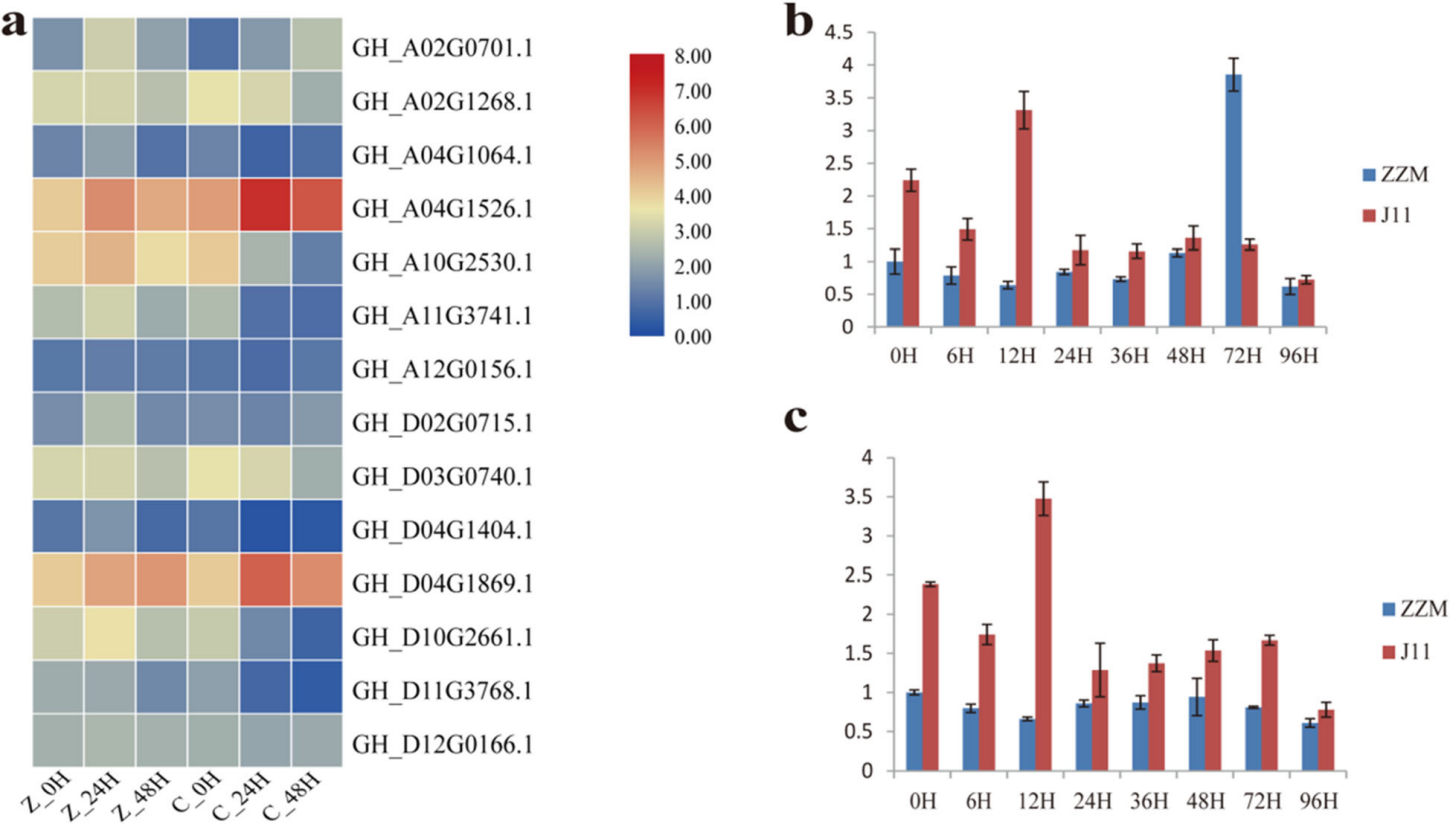
Expression profiling of *FBP* genes in response to *Verticillium* wilt stress. **a** Result of transcriptome analysis, Z represents the susceptible line CCRI36, C represents the resistant line MBI8255 and 0, 24 and 48 represent hours after inoculation with *V. dahliae*; **b** qRT-PCR results from *GH_A04G1526.1*; **c** qRT-PCR results from *GH_D04G1869.1*. The red bars represent Jimian 11 (J11) and the blue bars represent ZZM

In plant response to *Verticillium* wilt stress, the FPKM values of the *FBP* gene family members that were extracted from the previously mentioned transcriptome data showed that the homologous genes *GH_A04G1526.1* and *GH_D04G1869.1* had much higher expression values at 24 and 48 h after inoculation (HAI) with *Verticillium dahliae*, with their highest peaks being reached at 24 HAI (Fig. 7a, supplementary file 4). These results suggested a certain biological function of *FBP* genes in plant responses to *Verticillium* wilt stress.

The results of qRT-PCR analysis showed that both *GH_A04G1526.1* and *GH_D04G1869.1* had different expression behaviors in root tissues between susceptible and resistant cultivars at different developmental stages of *V. dahliae* after inoculation. In the VW tolerant cultivar Jimian 11(J11), both *GH_A04G1526.1* and *GH_D04G1869.1* had immediate responses to inoculation with *V. dahliae* and their expression levels reached a maximum at 12 HAI. The levels then dropped rapidly and maintained fairly low expression levels (Fig. 7b and c). In the VW susceptible cultivar ZZM, *GH_A04G1526.1* and *GH_D04G1869.1* acted differently, with *GH_A04G1526.1* slightly increasing its expression after inoculation up to 48 HAI, followed by its expression increasing rapidly and reaching a peak at 72 HAI (Fig. 7b), while *GH_D04G1869.1* maintained low expression throughout the entire experimental procedure (Fig. 7c). These different responses suggested that *GH_A04G1526.1* might take part in resistant reactions, while *GH_D04G1869.1* participated in susceptible reactions to *Verticillium* wilt in cotton.

**Fig. 8:**
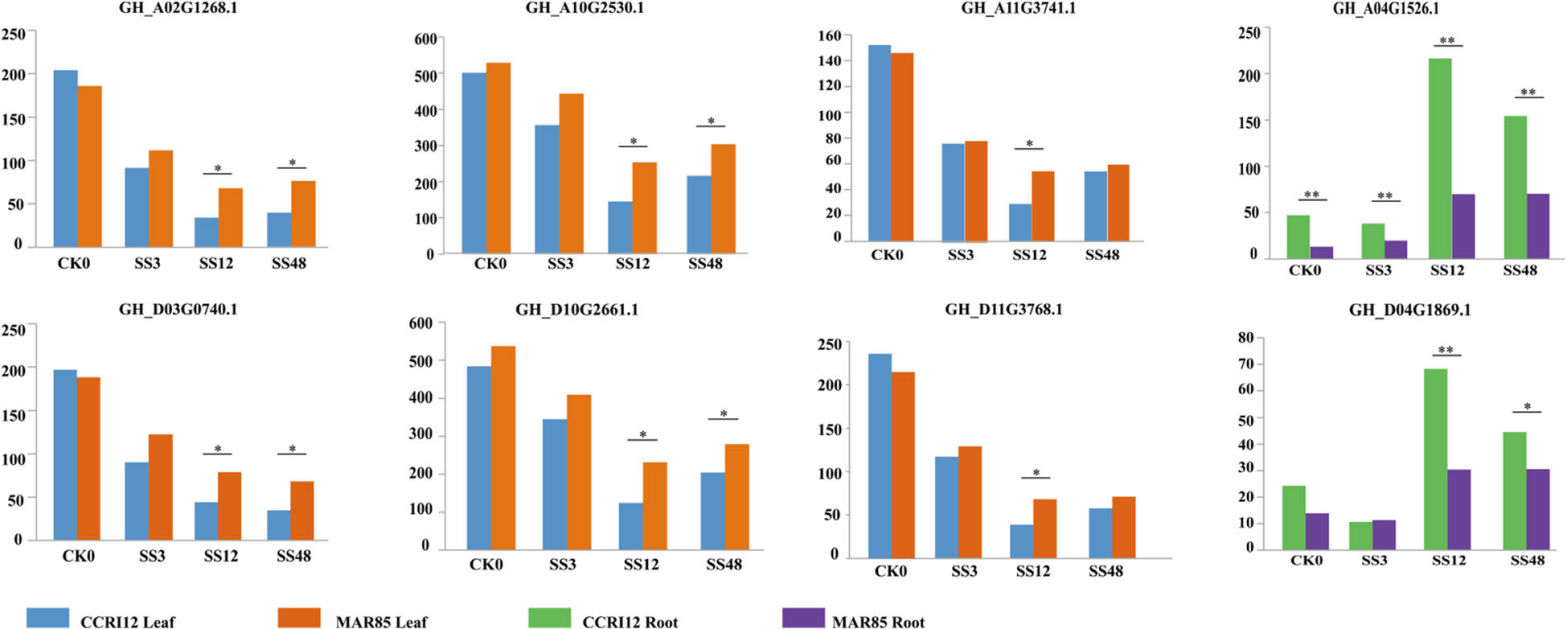
Expression profiling of upland cotton *FBP* genes evaluated using RNA transcriptome data analysis under salt stress

The responses of *FBP* genes to salt stress were also evaluated using RNA transcriptome data analysis [46] under salt stress (Fig. 8, supplementary file 4). Our transcriptome analysis indicated that six members of the *FBP* family, *GH_A10G2530.1*, *GH_D10G2661.1*, *GH_A11G3741.1*, *GH_D11G3768.1*, *GH_A02G1268.1*, and *GH_D03G0740.1*, had significantly higher responsive expression to salt stress treatments in foliage and two members, *GH_A04G1526.1* and *GH_D04G1869.1*, had significantly higher responsive expression in roots (Fig. 8). In the salt susceptible cultivar CCRI12, the tested genes that had expressions in foliage had similar expression tendencies in responses to salt pressure. Their expressions were significantly inhibited within 3 h after salt stress was imposed. This inhibition continued and reached its highest at 12 h after the initiation of stress. After this time, as time proceeded, the plant began to develop some sorts of “adaption” mechanisms, and their expression recovered to a certain level. In the salt tolerant semi-wild species MAR85, the inhibition of these genes was to a much smaller extent. It could be seen from our results that the expression levels of these genes at 12 h from salt resistant material were almost double those from the salt sensitive materials. These expression differences between two cultivars reached significant level at least in one treatment stage (Fig. 8). Both *GH_A04G1526.1* and *GH_D04G1869.1* had significant higher responsive expressions in root tissues of CCRI12 than in root tissues of MAR85 (Fig. 8).

## Discussion

### Cotton *FBP* genes identification

FBPase decomposes fructose 1,6-diphosphate to 6-phosphate fructose and inorganic phosphate. It provides a key substrate for sucrose synthesis in the cytoplasm, participates in the regulation of the sucrose synthesis pathway, and affects the distribution of carbohydrates. cpFBP is mainly involved in the Calvin cycle and the synthesis of starch [47], and cyFBP is mainly involved in the synthesis of sucrose [3]. Studies have revealed that two reactions with FBP isozymes are in the branching points from which their metabolites flow from the Calvin cycle into the process of either starch or sucrose synthesis. In *A. thaliana*, an *FBP* mutant resulted in the decrease in soluble sugar content and starch accumulation, and a significant increase in SOD activity [48]. The mutant also led to developmental changes including an increase in the number of root vascular bundles [48]. The cloning of the *FBP* gene has also been reported in several plant species including cotton, but there has been no further report in cotton regarding *FBP* genes or gene family.

Our study suggested that the *FBP* gene family maintained a certain stability in different diploid species including *A. thaliana* (in which four *FBP* genes were identified), *T. cacao* (five *FBP* genes), *G. arboretum* (seven *FBP* genes), and *G. raimondii* (seven *FBP* genes). There were no identified tandem repeats or segmental duplications in *FBP* family across these species (Figs. 1 and 4). The fluctuation of the environment forces organisms living in it to continuously evolve to adapt to changes [49]. Gene duplication is a basic process in genome evolution [50]. A duplicated gene may face several evolutionary fates, either becoming degraded by loss of function mutations, shared due to gene dosage effects or becoming sub−/neo-functionalized [50]. Yet the exact evolutionary events functioning on a gene are always episodic [49]. Polyploidization is one major episodic evolutionary event that can provide whole genome level genetic variation for plant adaptive evolution [51]. Even though the mechanisms of how polyploidy genomes establish their genetic structure are still unclear, such duplicated genes in polyploidy species may face the same evolutionary fates [50]. Our *FBP* collinearity analysis suggested that there was no tandem or segmental duplications in this *FBP* gene family in *Gossypium* species, despite the whole genome replication event due to polyploidization, which gave rise to the allotetraploid AD genome species (*G. hirsutum* and *G. barbadense*) from a natural hybridization between ancestors of *G. arboreum* or *G. herbaceum* (A group) and *G. raimondii* (D group) 1.7 to 1.9 million years ago [38, 40, 52, 53]. Afterwards, these species, which were involved in this hybridization event, independently evolved and were domesticated in different geographical areas [40]. Taken the findings in the current study that this *FBP* gene family was subject to purifying selection pressure based on their lower Ka/Ks values and that the high similarities in gene structures and *cis* regulatory elements in the 5′ upstream regions of each subgroup of the *FBP* gene family, we concluded that the *FBP* gene family in *Gossypium* genomes was evolutionarily conserved.

A noticeable fact of the collinearity analysis in our study was that some potential collinearity of *FBP* gene pairs between *G. barbadense* and *G*. *raimondii*, between *G. barbadense* and *G. arboreum* and between *G. hirsutum* and *G*. *raimondii*, were lost (Fig. 4c). These findings were supported by their independent evolutions after phylogenesis of each species. These results also implied that the *FBP* gene members in *G. barbadense* may have undergone a faster evolution than those on the rest of the *Gossypium* species in this study. This result was highly consistent with previous findings [54–56]. Moreover, we also demonstrated additional structural rearrangements in the A_t_ subgenome rather than in the D_t_ subgenome of *G. hirsutum* [55]. Evidence showed asymmetric subgenome domestication for directional selection towards fiber length [56]. *NAC* family genes in the A_t_ and D_t_ subgenomes displayed asymmetric molecular evolution in terms of gene loss rates, evolutionary rates, and expression levels [54]. Recent evidence has demonstrated a faster evolution rate in allotetraploid cotton than in diploid cotton, in the A_t_ subgenome rather than in the D_t_ subgenome, and in *G. barbadense* than in *G. hirsutum* [40].

Evidence has demonstrated that both *G. hirsutum* and *G. barbadense* were from one transoceanic hybridization event between two ancestors of diploid species *G. arboreum* or *G. herbaceum* and *G. raimondii* [52, 53]. Through evolutionary analysis of the genomic sequence, we also domonstrated that after diploid cotton differentiated from a common ancestor of cocoa, and five or more genome-wide replication events occurred [38]. Actually, although this allotetraploid cotton species had conserved gene order and collinearity between the A and D subgenomes [24], there were at least 9 translocations and 28 inversions identified in *G. hirsutum* draft genome [38]. A recent study confirmed these translocations between the A genome and the D genome in diploid cotton [40]. Previous comparative studies demonstrated that, in *Gossypium*, chromosomes D03 of *G. raimondii*, A02 of *G. arboreum*, GhA_t_03 of *G. hirsutum*, and GbA_t_03 of *G. barbadense* were homologous [40]. A reciprocal translocation event between chromosome A01 and A02 in diploid *G*. *arboretum* was estimated before the ancestor of the extent domesticated tetraploid cotton was formed [38, 40]. Our results revealed that the genes *Gorai.002G055400.1* in D02, *Ga02G0778.1* in A02, *GH_A02G1268.1* in GhA_t_02, *GH_D03G0740.1* in GhD_t_03, *GB_A02G1288.1* in GbA_t_02, and *GB_D03G0699.1* in GbD_t_03 were homologous genes (Fig. 4). This fact demonstrated that the gene *Ga02G0778.1* in A02 was located on a DNA fragment that was translocated from A01 to A02 (Fig. 6a, b). How these translocations affect the genes expression patterns is theretofore still unknown. We did, however, demonstrate a significant expression profiling difference between these orthologous genes regardless of whether they were on the translocated fragment.

### Functional biodiversity of *FBP* genes in *Gossypium* species

FBPs in our study were mainly sorted into two groups based on their subcellular compartmentalization, which conditioned their respective functions and regulatory mechanisms [7]. cpFBP is a key carbon-metabolizing enzyme that catalyzes the conversion of fructose-1,6-bisphosphate to fructose-6-phosphate in the Calvin cycle and eventually to starch, while cyFBP a key enzyme that catalyzes the conversion of triose phosphates to sucrose in the sucrose-biosynthesis pathway [7]. In the animal kingdom, FBP was found to be involved in various disease reactions, while in plants, it has been demonstrated to be involved in sugar partitioning [7], and thus, participates in plant growth [15, 17], as well as plant responses to biotic and abiotic stresses [14]. In the current study, we identified elevated expression of *FBP* genes during *Gossypium* fiber development (Fig. 6), in response to salt treatment (Fig. 8), and in response to *V. dahliae* inoculation (Fig. 7).

Cotton fibers are formed by a single ovule outer bead epidermal cell through a specialized process of initial differentiation, elongation, thickening, and dehydration to form mature epidermal fibers [19, 52, 57, 58]. The onset of cotton fiber differentiation usually occurs near the day of flowering (0 DPA). These initially differentiated fibrocytes develop into fiber cells. The secondary expansion of epidemic cells is initiated after 7 to 10 DPA, and these cells gradually develop into fuzz that is attached to the surface of a seed [57–59]. Factors that influence the initiation, differentiation, and development of fibers include phytohormones, such as IAA and GA, and brassinolactone, as well as transcription factors [60–63]. The arrangement of cellulose deposition plays an important role in the formation of fiber strength and fineness [64, 65]. It has been demonstrated that the sucrose content in the developing fibers of immature-fiber-mutants are significantly lower than that of TM-1 [66]. This could be explained by the finding that sucrose, which is imported into developing fiber cells, is the major carbon source for cellulose synthesis and energy production there. Sucrose, together with K^+^ and malate, also provides the major active osmotic solutes required to maintain cell turgor for fiber elongation [30]. It has also been demonstrated that suppressing *GhSCP2D* expression in cotton fiber eventually activates sucrose transporter genes such as SUTs and SWEETs in early fiber developmental stages [67]. The transcription factor GhMYB212 was also demonstrated to be involved in sucrose transport into elongating fiber cells [26]. A sufficient and sustainable supply of this sugar is essential for fiber development. Here we demonstrated high expression of some *cyFBP* genes, including *GH_A02G0701.1, GH_D02G0715.1*, and *GH_A02G1268.1*, in developing cotton fibers, which hinted that these *cyFBP* genes probably assumed the responsibility of maintaining the supply of sucrose in developing fiber cells, especially during late secondary cell wall thickness stages.

*Verticillium* wilt is the most important disease restricting cotton production due to its serious impacts on cotton yield and fiber quality [68]. Bioinformatic analysis revealed that the 5′-upstream regulatory regions of *cyFBP* genes in the current study, in particular in *GH_A04G1526.1* and *GH_D04G1869.1* (Fig. 3), harbor pathogen-induced, *cis*-acting elements, including W-boxes, GCC-motifs, TC-rich repeats, JERE-motifs, Gstl-motifs and D-motif components, G boxes, GT-1, GCC boxes and S boxes [69–72]. These findings hinted that these cyFBP genes affect a certain impact on plant responses to the onset of *Verticillium* wilt. It is generally recognized that the responses of cotton plant to *Verticillium* wilt are mainly accomplished by thickening the cell wall and blocking the catheter, which includes gel formation, epidermal lignification, and internal tissue cork modification to form an invasion plugging body, which prevents the spread of the pathogen in the plant and confines the invading *V. daliae* to a restricted local area [73]. After infection with *V. daliae*, corresponding changes in cotton plant metabolism also include the biosynthetic enhancement of lignin, phytoalexin, tannin, polysaccharide, gossypol, and lipids [70, 74]. Lignification of the cell wall enhances its mechanical resistance to the penetration of fungal appressoria [75]. The lignin-based disease resistance mechanisms are very important in the processes of plant pathogenic microorganism interactions [75]. Lignification has been extensively studied in cotton resistance [76–78], and tolerance to salt stress [79]. Actually, evidence suggests that increasing metabolizable sugar content should positively influence the abundance of lignins. Researchers have speculated that there also may be a mechanism in plants that senses changes in sugar content and regulates lignin biosynthesis [80].

In the current study, we identified significant differences in the inhibition of both *cpFBP* and *cyFBP* gene expression levels between CRI12, an upland cotton cultivar, and MAR85, a semi-wild relative of upland cotton (Fig. 8). Previous studies revealed that a *cpFBP* gene from *Oriza sativa* was more sensitive to salt stress treatments than that from its close halophytic relative, *Porteresia coarctata* [81, 82]. The *cpFBP* genes from the two plants were determined to have five amino acid residue differences [83]. Xiao et al. [14] detected a significant inhibition of *PhcpFBP* gene expression at the early stages of high temperature stress, while with persistent high temperature stress, the expression of *PhcpFBP* gradually resumed a normal level. Hark et al. [84] determined that the *cyFBP* gene was not expressed continuously in severe water stress, although a low level of enzyme activity was still present (33% of control). These studies indicated that both the *cpFBP* and *cyFBP* genes might be involved in plant responses to salt stress in one way or another. At the same time, the genes that have elevated expressions in response to inoculation with *V. daliae* also showed responsive expression to salt treatment (Fig. 8). This implies that the pathways these genes catalyze might be correlated to plant responses to both salt stress and *V. daliae* inoculation.

## Conclusions

In this study, a total of 41 *FBP* genes were identified in four cotton species, distributed to six chromosomes in diploid cotton and 11 chromosomes in tetraploid cotton, and could be classified into two classes. There were large translocations between A02 and A03 chromosomes in tetraploid cotton and there was an inversion translocation on the A02 and D02 homologous chromosomes in diploid cotton, meaning the collinearity of the *FBP* genes changed. Through our results from RNA-seq and qRT-PCR, we know that the different *FBP* genes had different functions, not only affecting fiber development, but also the response to *Verticillium* wilt and salt stress. Additionally, GARE-motif may affect gene expression during the fiber development. Genes of the same subclass have the same motif, gene structure, and similar *cis*-acting elements, meaning they have similar functions. All of these results provide a foundation for further study on the function of the *FBP* genes in cotton fiber development and environmental adaptability.

## Methods

### Plant growth and tissue sampling

#### Fiber samples

The *G. hirsutum* cultivars ZR014121 (The National Cotton Germplasm Medium-term Bank of China, accession number ZM115357) and EZ60 (The National Cotton Germplasm Medium-term Bank of China, accession number M116025) were grown in the experimental farm of the Institute of Cotton Research, Chinese Academy of Agricultural Sciences in Anyang, Henan, China. The experimental cultivars were planted in a scale of ten rows with row-length of 5 m, row-spacing of 0.8 m and plant-spacing of 0.2 m. Three repeats for each cultivar were planted. The flowers were tagged on the day of anthesis and that day was regarded as 0 DPA. Flowers and developing bolls were sampled on 0, 5, 10, 15, 20, 25, and 28 DPA from the tagged flowers and the ovules and developing fibers were harvested from the developing bolls and samples were immediately dipped in liquid nitrogen and stored at − 80 °C for future use.

#### Root samples after *Verticillium dahliae* inoculation

The *G. hirsutum* cultivars Jimian 11 (J11) (The National Cotton Germplasm Medium-term Bank of China, accession number M110861), which is tolerant to *V. dahliae*, and ZZM (The National Cotton Germplasm Medium-term Bank of China, accession number M115705), which is susceptible to *V. dahlia*, were used in the study. The plant materials were grown in the green house in the Institute laboratory in three repeats. For each repeat, five uniform seedlings were maintained in each treatment. At the two-leaf stage, the cotton plants were inoculated with strain vd080 of *V. dahliae* by root-dips. Roots were sampled at 0 h (0 H), 6 H, 12 H, 24 H, 36 H, 48 H, 72 H, and 96 H after inoculation. Three replicates were collected and immediately stored at − 80 °C.

#### Transcriptome analyses and quantitative real-time PCR

Transcriptome data of cotton fiber development of *G. hirsutum* TM-1 and *G. barbadense* Hai7124 were downloaded (accession number PRJNA490626, https://www.ncbi.nlm.nih.gov/bioproject/ PRJNA490626) from the Sequence Read Archive (SRA) of the NCBI database (https://www.ncbi.nlm.nih.gov/) [40]. Raw read data (Sra) were converted to Fastq data using Fastq-dump, and Fastq data were filtered using Trimmomatic-0.36 to remove residues and low quality reads. The filtered data were then assembled from scratch using Trinity, the transcripts were assembled, and the reads were then posted back to the reference genome to calculate expression. After FPKM values were obtained, a heat map was generated using MEV 4.0 software [85].

The expression levels of the members of the *G. hirsutum FBP* gene family were extracted and re-analyzed from the transcriptome data of MBI8255 and CCRI36 after *Verticillium dahliae* inoculation using high-throughput sequencing methods described in a previous study [86]. The transcriptome data from CRI12 and MAR85 in salt stress were kindly provided by Dr. Liu’s Lab [46]. The expression heat map of each member of the *G. hirsutum FBP* gene family was created using MEV 4.0 software [85].

Total RNA samples of cotton fiber and roots were extracted using an RNAprep Pure Plant Kit from Tiangen Biotechnology Co., Ltd. (Beijing, China) and were visualized on 1% agarose gels. cDNA was synthesized using a FastQuant RT Kit (Tiangen Biotech (Beijing) Co., Ltd.). Primer premier 5.0 software was used to design gene specific primers (Supplementary file 5) for fluorescent quantitative PCR based on full-length gene cDNA. The GhHistone3 gene used as an internal reference gene was downloaded from the NCBI Nucleotide database (accession number AF024716). qRT-PCR was performed using a LightCycler® 480 II Real-Time PCR Instrument (Basel Roche, Switzerland). The expression levels of the genes were calculated using the 2^-ΔΔCT^ method, with three independent PCR amplifications [87].

#### Identification of cotton *FBP* genes

The latest versions of the genomes of four cotton species were downloaded from CottonFGD (https://cottonfgd.org/) [88], including *G. hirsutum* (https://cottonfgd.org/about/download/assembly/genome.Ghir.ZJU.fa.gz), *G. barbadense* (https://cottonfgd.org/about/download/assembly/genome.Gbar.ZJU.fa.gz), *G. arboreum* (https://cottonfgd.org/about/download/assembly/genome.Garb.CRI.fa.gz), *G. raimondii* (https://cottonfgd.org/about/download/assembly/genome.Grai.JGI.fa.gz). Some of the genomes for comparative analysis, including *T. cacao* (Tcacao/v2.1, https://genome.jgi.doe.gov/portal/pages/dynamicOrganismDownload.jsf?organism=Tcacao#) [89], *Oryza sativa* (Osativa/v7.0, https://genome.jgi.doe.gov/portal/pages/dynamicOrganismDownload.jsf?organism=Osativa#) [90], *A. thaliana* (Athaliana/TAIR10, https://genome.jgi.doe.gov/portal/pages/dynamicOrganismDownload.jsf?organism=Athaliana#) [91], *P. trichocarpa* (Ppatens/v3.3, https://genome.jgi.doe.gov/portal/pages/dynamicOrganismDownload.jsf?organism=Ptrichocarpa#) [92], *V. vinifera* (Vvinifera/v2.1, https://genome.jgi.doe.gov/portal/pages/dynamicOrganismDownload.jsf?organism=Vvinifera#) [93]*, S. moellendorffii* (Smoellendorffii/v1.0, https://genome.jgi.doe.gov/portal/pages/dynamicOrganismDownload.jsf?organism=Smoellendorffii#) [94], *Z. mays* (ZmaysPH207/v1.1, https://genome.jgi.doe.gov/portal/pages/dynamicOrganismDownload.jsf?organism=ZmaysPH207#) [95]*, G. max* (Gmax/Wm82.a4.v1, https://genome.jgi.doe.gov/portal/pages/dynamicOrganismDownload.jsf?organism=Gmax#) [96], and *P. patens* (Ppatens/v3.3, https://genome.jgi.doe.gov/portal/pages/dynamicOrganismDownload.jsf?organism=Ppatens#) [97]*,* were downloaded from the ePhytozome V12.1 database (http://www.phytozome.net). A hidden Markov model (HMM) profile (PF00316) was downloaded from EMBL-EBI (http://pfam.xfam.org/family/PF00316/hmm). The hmmsearch program in HMMER 3.0 software [98] was then used to search for protein sequences of these four *Gossypium* species using Ces.hmm as a model and --cut_ga as a parameter. The relative molecular weight and the theoretical isoelectric point prediction of the obtained amino acid sequences of FBP proteins were performed using the ExPASy (http://expasy.org/) online database.

#### *FBP* gene family system evolution, gene structure, and chromosomal distribution analysis

Multi-sequence alignments of all FBP protein sequences were performed using Clustal X [99], and phylogenetic trees of related proteins were constructed using the MEGA software proximity method (version 6.06) (Neighbor-Joining, NJ) [100], with the calibration parameter Bootstrap being set to 1000. The structure of the candidate genes was analyzed using the online software GSDS 2.0 (http://gsds.cbi.pku.edu.cn/) [101]. The MEME online tool (http://meme-suite.org/) was used to analyze the conserved motifs of the amino acid sequences (Motif), in which the parameter settings were as follows: the minimum length of the conserved motif was six, the maximum length was fifty, and the maximum motif number was ten.

### Collinearity and selection pressure analysis

To determine whether the *FBP* gene family expanded through segmental duplication or tandemduplication events, a collinear analysis was completed with an all-to-all BLAST array (*E*-value of 1e-5) in the MCScan program [102]. Selection pressure analysis was performed by calculation of the Ka (non-synonymous substitution rate) and Ks (synonymous substitution rate) values of homologous genes using KaKs_Calculator 2.0 software [103].

### Prediction of *cis*-acting elements in cotton FBP gene promoters

Two thousand bp upstream sequences of the candidate genes were extracted from the genome sequences of the four *Gossypium* species. *Cis*-acting elements were predicted using online software PLANT CARE (http://bioinformatics.psb.ugent.be/webtools/plantcare/Html/) [104].

## Supplementary information

**Additional file 1 **Multiple sequence alignment involving 114 sequences from the genomes of the following eight species: *G. hirsutum*, *G. arboreum*, *G. barbadense*, *G. raimondii*, *A. thaliana*, *T. cacao*, *P. trichocarpa*, *G. max*, *Z. mays, V. vinifera*, *S. moellendorffii, P. patens,* and *O. sativa*.

**Additional file 2.** Amino acid compositions of FBP genes.

**Additional file 3 **Ka/Ks ratio of *FBP* homologous genes.

**Additional file 4 **The FPKM value of *FBP* genes in tetraploid cotton species.

**Additional file 5.** The primer sequences used for qRT-PCR.

**Additional file 6 Figure S1.** Logos of 10 motifs for four cotton species according to the MEME suite.

## Data Availability

The latest versions of the genome of four cotton species were downloaded from CottonFGD (https://cottonfgd.org/), including *G. hirsutum* (https://cottonfgd.org/about/download/assembly/genome.Ghir.ZJU.fa.gz), *G. barbadense* (https://cottonfgd.org/about/download/assembly/genome.Gbar.ZJU.fa.gz), *G. arboreum* (https://cottonfgd.org/about/download/assembly/genome.Garb.CRI.fa.gz), *G. raimondii* (https://cottonfgd.org/about/download/assembly/genome.Grai.JGI.fa.gz). Some of the genomes for comparison analysis, including *T. cacao* (Tcacao/v2.1, https://genome.jgi.doe.gov/portal/pages/dynamicOrganismDownload.jsf?organism=Tcacao#), *O. sativa* (Osativa/v7.0, https://genome.jgi.doe.gov/portal/pages/dynamicOrganismDownload.jsf?organism=Osativa#), *A. thaliana* (Athaliana/TAIR10, https://genome.jgi.doe.gov/portal/pages/dynamicOrganismDownload.jsf?organism=Athaliana#), *P. trichocarpa* (Ptrichocarpa/v3.1, https://genome.jgi.doe.gov/portal/pages/dynamicOrganismDownload.jsf?organism=Ptrichocarpa#), *V. vinifera* (Vvinifera/v2.1, https://genome.jgi.doe.gov/portal/pages/dynamicOrganismDownload.jsf?organism=Vvinifera#), *S. moellendorffii* (Smoellendorffii/v1.0, https://genome.jgi.doe.gov/portal/pages/dynamicOrganismDownload.jsf?organism=Smoellendorffii#), *Z. mays* (ZmaysPH207/v1.1, https://genome.jgi.doe.gov/portal/pages/dynamicOrganismDownload.jsf?organism=ZmaysPH207#), *G. max* (Gmax/Wm82.a4.v1, https://genome.jgi.doe.gov/portal/pages/dynamicOrganismDownload.jsf?organism=Gmax#) and *P. patens* (Ppatens/v3.3, https://genome.jgi.doe.gov/portal/pages/dynamicOrganismDownload.jsf?organism=Ppatens#) were downloaded from the ePhytozome V12.1 database (http://www.phytozome.net).Transcriptome data of cotton fiber development of *G. hirsutum* TM-1 and *G. barbadense* Hai7124 was downloaded (accession number PRJNA490626, https://www.ncbi.nlm.nih.gov/bioproject/PRJNA490626) from the NCBI BioProject database (https://www.ncbi.nlm.nih.gov/)”. The GhHistone3 gene dataset was downloaded from the NCBI Nucleotide database (accession number AF024716,https://www.ncbi.nlm.nih.gov/nuccore/AF024716). All extra data generated or analyzed during this study are included in this published article.

## Abbreviations

aa: Amino acid
DPA: Day post anthesis
Ga: *Gossypium arboreum*
Gb: *Gossypium barbadense*
Gh: *Gossypium hirsutum*
Gr: *Gossypium raimondii*
Ka: Nonsynonymous substitution rate
Ks: Synonymous substitution rate
FBP: Fructose-1,6-bisphosphatase
qRT-PCR: Quantitative real-time polymerase chain reaction
SPS: Sucrose phosphate synthase
