# Supplementary figures and images for: Disequilibrium evolution of the Fructose-1,6-bisphosphatase gene family leads to their functional biodiversity in Gossypium species
Source: BMC Genomics. 2020 Jun 1;21:379. doi: 10.1186/s12864-020-6773-z (PMC7262775; doi:10.1186/s12864-020-6773-z)

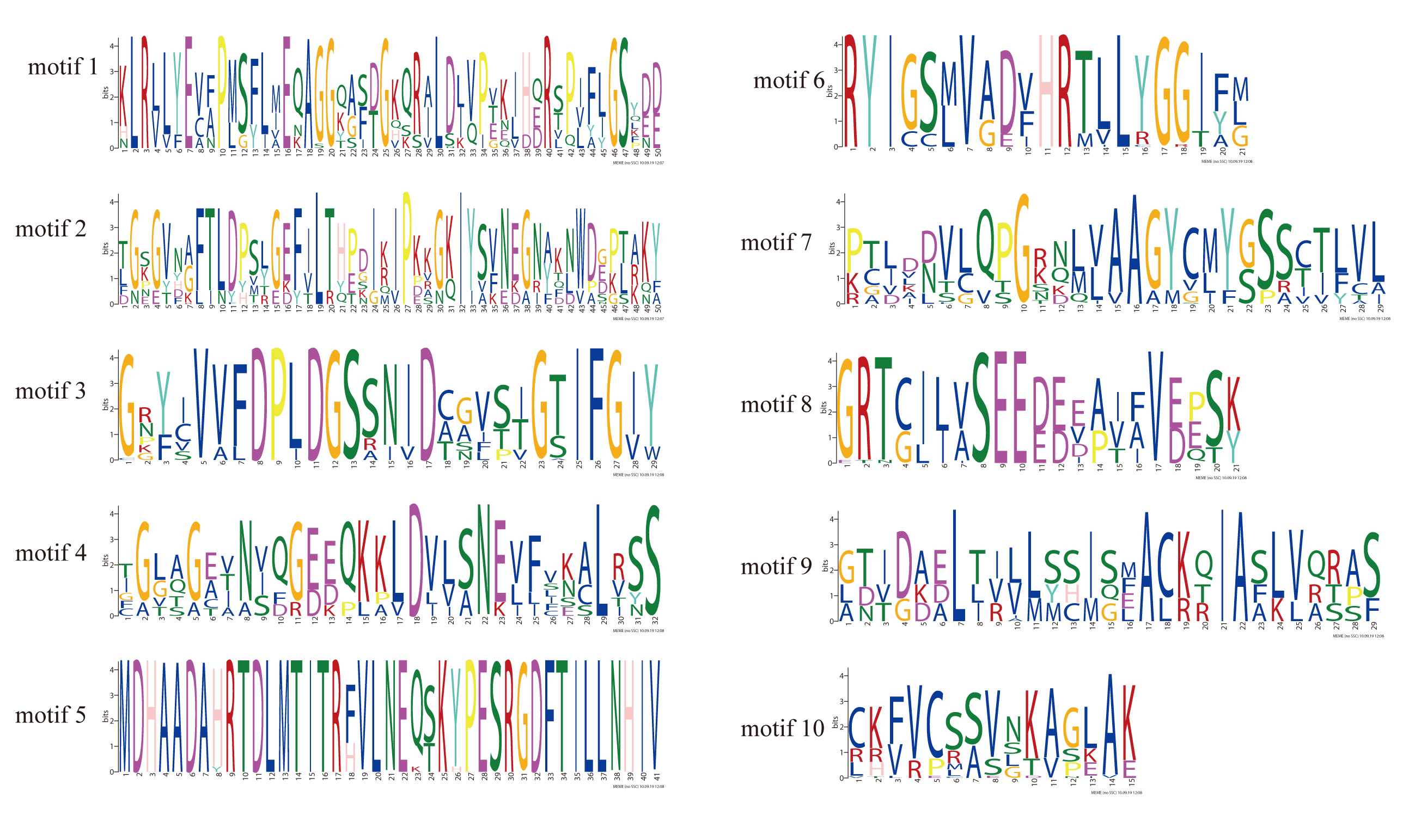

Supplement: Supplementary file 6 — Additional file 6 Figure S1. Logos of 10 motifs for four cotton species according to the MEME suite. [file 12864_2020_6773_MOESM6_ESM.tif]
